# Human immunodeficiency virus type I-specific CD8^+ ^T cell subset abnormalities in chronic infection persist through effective antiretroviral therapy

**DOI:** 10.1186/1471-2334-10-129

**Published:** 2010-05-25

**Authors:** Julia Pohling, Katrin Zipperlen, Natasha A Hollett, Maureen E Gallant, Michael D Grant

**Affiliations:** 1 Immunology and Infectious Diseases Program, Division of BioMedical Sciences, Faculty of Medicine, Memorial University of Newfoundland, St. John's NL, Canada

## Abstract

**Background:**

Effective highly active antiretroviral therapy (HAART) reduces human immunodeficiency virus (HIV) replication, restores CD4^+ ^T lymphocyte counts and greatly reduces the incidence of opportunistic infections. While this demonstrates improved generalized immune function, rapid rebound to pre-treatment viral replication levels following treatment interruption indicates little improvement in immune control of HIV replication. The extent to which HAART can normalize HIV-specific CD8^+ ^T cell function over time in individuals with chronic infection remains an important unresolved issue. In this study, we evaluated the magnitude, general specificity and character of HIV specific CD8^+ ^T cell responses at four time points across 2-9 years in 2 groups of chronically infected individuals separated on the basis of either effective antiretroviral suppression or ongoing replication of HIV.

**Methods:**

Peripheral blood mononuclear cells (PBMC) were stimulated with overlapping 15mer peptides spanning HIV Gag, Pol, Env and Nef proteins. Cells producing interferon-γ (IFN-γ) or interleukin-2 (IL-2) were enumerated by ELISPOT and phenotyped by flow cytometry.

**Results and Conclusions:**

The magnitude of the HIV-specific CD8^+ ^T cell response ranged from < .01 to approximately 1.0% of PBMC and was significantly greater in the group with detectable viral replication. Stronger responses reflected higher numbers of CD8^+^CD45RA^- ^effector memory cells producing IFN-γ, but not IL-2. Magnitude, general specificity and character of the HIV-specific CD8^+ ^T cell response changed little over the study period. While antiretroviral suppression of HIV in chronic infection reduces HIV-specific CD8^+ ^T cell response magnitude in the short term, it had no significant effect on response character over periods up to 9 years.

## Background

Human immunodeficiency virus (HIV) infection progressively impairs immune function, including that of the HIV-specific CD8^+ ^T cells that partially control HIV replication. This impairment manifests in phenotypic and functional changes affecting key CD8^+ ^T cell properties such as proliferation, differentiation, cytokine production profile, signal transduction, and survival [1-8]. Compared to cytomegalovirus (CMV)-specific CD8^+ ^T cells, HIV-specific CD8^+ ^T cells are highly skewed towards effector memory (Tem) status, suggesting reduced differentiation into CD45RA^+ ^terminal effectors (Ttd) [9]. In addition, limited HIV-specific CD8^+ ^T cell proliferation suggests defective generation and/or maintenance of central memory T cells (Tcm) [10]. Understanding the fate of these subset abnormalities in HIV-infected individuals on highly active antiretroviral therapy (HAART) is important for defining the need for complementary treatment and for gauging practical limits to immune recovery.

When effective, HAART increases CD4^+ ^T lymphocyte counts, reduces opportunistic infections and according to early studies, normalizes immune responses [11,12]. However, more specific analyses have recently raised doubt as to whether HIV-specific CD4^+ ^and CD8^+ ^T cell responses recover characteristics normally associated with effective control of intracellular pathogens [13-18]. Early control of HIV replication may be required to preserve HIV-specific CD4^+ ^T cells and fully functional HIV-specific CD8^+ ^T cells appear peculiar to those long term non progressors (LTNP) who maintain low HIV replication levels without treatment [19]. Thus, a more pessimistic view is that the level to which HIV-specific T cell responses deteriorate prior to treatment represents an intractable deficit, insensitive to HAART-related immune recovery. This issue remains controversial as two recent studies reached diametrically opposite conclusions of clear HAART-related improvement versus no change [20,21]. These conflicting views could reflect different lenses, a broad range of pre-HAART deterioration in the groups studied and inherent human variation in thresholds beyond which the possibility or rate of recovery lessens.

In this study, we investigated evolution of the magnitude, specificity and memory subset distribution of HIV-specific CD8^+ ^T cell responses over a 2 to 9 year period in two groups: one with continuous suppression of HIV replication on HAART; and another with continuously detectable HIV replication. We reasoned that contrasting trajectories of the 2 groups would highlight any impact HAART was having towards reversal or prevention of HIV-specific CD8^+ ^T cell abnormalities and help resolve this critical issue.

## Methods

### Subjects

Study participants recruited through the St. John's General Hospital HIV Clinic, St. John's, Newfoundland, Canada were selected on the basis of ≥ 24 sequential months of either complete suppression of HIV replication below levels detectable by plasma virus load testing (< 2.6 or < 1.7 log_10 _copies HIV RNA/ml plasma by Roche ™Amplicor standard or ultrasensitive testing respectively) or ≥ 24 sequential months of detectable plasma virus loads. Subjects were grouped accordingly and at least 4 sequential PBMC samples, over a period as stipulated above, were selected for analysis. Other relevant immunological and virological characteristics of the study subjects were obtained from patient charts and noted accordingly in results. All participants provided informed consent for whole blood donation, immunological studies and researcher access to medical laboratory records. The Memorial University Faculty of Medicine Human Investigation Committee gave ethical approval for this study.

### Peripheral blood mononuclear cell (PBMC) isolation

Acid-citrate-dextrose treated whole blood obtained by forearm venipuncture at regular intervals concurrent with medically scheduled bloodwork was diluted 1:2 with phosphate buffered saline (PBS) and PBMC isolated by Ficoll-Hypaque (GE Healthcare Bio-Science) density gradient centrifugation. Samples were crypopreserved in lymphocyte medium consisting of RPMI 1640 with 10% fetal bovine serum (FBS), 100 IU/ml penicillin, 100 μg/ml streptomycin, 2 mM L-glutamine, 10 mM HEPES buffer solution and 2 × 10^-5 ^M 2-mercaptoethanol (all from Invitrogen) with FBS increased to 20% and supplemented with 10% dimethylsulfoxide (Sigma).

### ELISPOT Assays

Individual PBMC samples from 6 non-infected controls and series of 4 PBMC samples from 30 HIV-infected individuals in the 2 groups were stimulated with pools of overlapping 15mer peptide sets spanning HIV clade B Gag, Pol, Env and Nef (National Institutes of Health AIDS Reference Reagent Repository). Individual peptides were dissolved at 10 mg/ml in DMSO and then pooled in sets of 49 (Nef), 123 (Gag), 125 (Pol1), 123 (Pol2), 128 (Env1), and 83 (Env2) in unsupplemented RPMI at a final concentration of 1.0 μg/ml of each peptide. Samples were thawed and cultured overnight before counting to ensure accurate numbers of viable cells were used. For interferon-γ (IFN-γ) assays, microtitre plates (Multiscreen; Millipore) were coated overnight at 4°C with 100 μl of 7.5 μg/ml anti-IFN-γ monoclonal antibody (mAb) 1-D1K (Mabtech) and then washed 3 times with PBS. For interleukin-2 (IL-2) assays, plates were coated overnight at 4°C with 100 μl of 15 μg/ml anti-IL-2 mAb IL-2-/249 (Mabtech) and then washed 3 times with PBS. Cells were added at 4.0 or 1.0 × 10^5^/well in 100 μl medium in single or dual replicates for IL-2 and IFN-γ detection respectively. Peptides (20 μl/pool) were added to a final concentration of 0.1 μg/ml with the volume in each well adjusted to 200 μl with medium. After overnight (16 hr) incubation, the plates were washed 6 times as above and 100 μl of 1 μg/ml biotinylated 7-B6-1 or IL-2-II (Mabtech) added for 2 h. Plates were then washed 6 times and 100 μl/well of a 1/1000 dilution of streptavidin-alkaline phosphatase (AP) (Mabtech) was added for 1 h. Plates were again washed 6 times and 100 μl/well of chromogenic AP susbstrate (BioRad) was added. After 30 min, plates were washed with tap water to stop reactions and then air-dried. Spots were counted with an Immunoscan reader (Celluar Technology Limited) and results reported as IFN-γ spot forming cells (sfc)/10^6 ^PBMC (when ≥ 50) or IL-2 sfc/10^6 ^PBMC (when ≥ 0) after subtraction of background (sfc/10^6 ^PBMC cultured overnight in medium alone).

### Flow cytometry

When sufficient cells were available, 1 × 10^6 ^PBMC were incubated overnight with each individual peptide pool or in medium alone as negative control under the same conditions as ELISPOT except for addition of 10 μg/ml brefeldin A (Sigma) after 1 h of incubation. If ELISPOT results indicated ≥ 500 sfc/10^6 ^PBMC, the corresponding samples and controls were processed for flow cytometry. Cells were washed with PBS plus 1% FCS and 5 mM EDTA, then surface stained with anti-CD8-PerCP (clone RPA-T8, Biolegend) and anti-CD45RA-PE (clone JS-83, eBioScience). After washing, cells were fixed, permeabilized (Dako intrastain), and stained with anti-IFN-γ-APC (clone 4S-B3, eBioScience). At least 200,000 events were acquired on a FacsCalibur flow cytometer (Becton Dickenson) and CD8^+ ^cells gated and analyzed for IFN-γ and CD45RA expression with Cellquest Pro software (Becton Dickenson).

### Statistical analysis

Statistical analysis was carried out with GraphPad Prism 4.03 Software. Normality of data distribution was assessed by the Kolmogrov-Smirnov test. When data was normally distributed, means were presented for comparison. If data did not fit a normal distribution, medians were presented for comparison. Repeated measures ANOVA was used to compare the frequency of HIV-specific CD8^+ ^T cells producing IFN-γ or IL-2 between and within groups at different time points and to compare between groups the percentage of CD45RA^+ ^HIV-specific CD8^+ ^T cells producing IFN-γ. Paired Student's *t *test was used to compare initial and final CD4^+ ^T lymphocyte counts and initial and final percentages of CD45RA^+ ^HIV-specific CD8^+ ^T cells producing IFN-γ within groups. Probability values < 0.05 were considered significant.

## Results

### Subjects' characteristics

General characteristics of the two HIV-infected groups prior to and over the course of the study are reported in table 1 (undetectable viral replication) and table 2 (detectable viral replication). All subjects were chronically infected for a documented duration of from at least 5 to > 15 years. Most individuals in both groups had endured relatively high levels of HIV replication and had progressed to advanced HIV infection at some point as illustrated by zenith viral loads and CD4 nadirs < 200/μL peripheral blood. Those in the uncontrolled replication group had plasma virus loads throughout the study course that were substantially lower than their zenith, suggesting partial efficacy of their antiretroviral therapy as the recorded zenith was not associated with acute infection. Three individuals (187, 195 and 209) in the uncontrolled infection group had never received antiretroviral therapy up to and through the course of this study, while all others in both groups had received HAART in the form of triple therapy with drugs of at least 2 classes. The documented duration of undetectable plasma virus load in the controlled infection group ranged from 3 to 84 months preceding this study.

**Table 1:** General characteristics and changes for subjects with undetectable HIV replication

ID	Duration of infection	^a^VL zenith	^b^CD4 nadir	^c^Duration of suppression	^d^CD4_i_	CD4_f_	^e^Months study course	^f^CD8^+ ^IFNγ^+ ^CD45RA^+^_i_	CD8^+ ^IFNγ^+ ^CD45RA^+^_f_
27	>15 yrs	5.13	308	21 months	702	1364	90	13%	26%
30	>15 yrs	4.53	314	84 months	630	1089	28	ND	ND
50	>15 yrs	5.45	18	7 months	220	627	108	52%	50%
62	>15 yrs	4.61	154	32 months	738	756	28	10%	15%
90	>15 yrs	6.22	73	24 months	280	352	96	79%	84%
109	>15 yrs	3.66	145	72 months	740	665	24	22%	16%
128	>12 yrs	4.53	364	3 months	570	912	66	44%	52%
134	>12 yrs	5.01	96	72 months	850	1020	24	ND	ND
143	>10 yrs	4.77	400	24 months	400	682	60	ND	22%
149	>9 yrs	5.67	9	36 months	338	264	24	ND	ND
155	>9 yrs	5.10	210	3 months	368	408	54	10%	10%
174	>7 yrs	5.53	380	12 months	494	768	30	ND	ND
176	>7 yrs	<2.6	245	12 months	361	750	36	ND	ND
193	>6 yrs	<2.6	40	12 months	405	324	24	23%	13%
200	>5 yrs	>5.88	192	3 months	567	957	24	ND	ND

^a^The highest recorded virus load (VL) for each subject in log_10 _copies HIV RNA/mL plasma.

^b^The lowest recorded peripheral blood CD4^+ ^T lymphocyte count for each subject.

^c^The length of time before analysis of HIV-specific CD8^+ ^T cell responses for which each subject experienced suppression of viremia below detectable levels.

^d^The number of CD4^+ ^T lymphocytes/μL peripheral blood for each subject at beginning (CD4_i_) and end (CD4_f_) of period over which HIV-specific CD8^+ ^T cell responses were assessed.

^e^Total time period over which four assessments of HIV-specific CD8^+ ^T cell responses occurred.

^f^Percentage of HIV-specific CD8^+ ^T cells producing INF-γ that expressed CD45RA at beginning (_i_) and end (_f_) of period over which HIV-specific CD8^+ ^T cell responses were assessed.

**Table 2:** General characteristics and changes for subjects with detectable HIV replication

ID	Duration of infection	^a^VL zenith	^b^CD4 nadir	^c^VL study course	^d^CD4_i_	CD4_f_	^e^Months study course	^f^CD8^+ ^IFNγ^+ ^CD45RA^+^_i_	CD8^+ ^IFNγ^+ ^CD45RA^+^_f_
11	>15 yrs	>5.88	63	4.58	308	335	60	15%	16%
17	>15 yrs	4.48	296	4.12	518	490	30	ND	ND
35	>15 yrs	4.77	176	3.37	660	736	48	35%	12%
39	>15 yrs	5.64	24	2.70	754	961	30	6%	14%
51	>15 yrs	5.61	40	3.29	390	297	36	33%	25%
57	>15 yrs	5.48	176	4.25	420	506	36	14%	8%
77	>15 yrs	5.00	207	3.80	692	288	24	ND	ND
78	>15 yrs	>5.88	32	4.53	110	184	36	24%	23%
85	>14 yrs	4.60	307	2.65	684	663	66	11%	11%
98	>13 yrs	5.73	69	3.79	484	435	48	ND	ND
116	>12 yrs	5.56	44	3.22	319	378	48	23%	23%
125	>12 yrs	5.06	59	4.52	496	209	42	31%	ND
187	>7 yrs	3.93	1064	2.89	1127	1419	36	37%	ND
195	>5 yrs	4.93	462	4.54	518	462	24	27%	25%
209	>5 yrs	4.97	500	4.72	812	500	24	ND	ND

^a^The highest recorded virus load (VL) for each subject in log_10 _copies HIV RNA/mL plasma.

^b^The lowest recorded peripheral blood CD4^+ ^T lymphocyte count for each subject.

^c^Virus load (log_10 _copies HIV RNA/mL plasma) averaged over the 4 time points for each subject.

^d^The number of CD4^+ ^T lymphocytes/μL peripheral blood for each subject at beginning (CD4_i_) and end (CD4_f_) of period over which HIV-specific CD8^+ ^T cell responses were assessed.

^e^Total time period over which four assessments of HIV-specific CD8^+ ^T cell responses occurred.

^f^Percentage of HIV-specific CD8^+ ^T cells producing INF-γ that expressed CD45RA at beginning (_i_) and end (_f_) of period over which HIV-specific CD8^+ ^T cell responses were assessed.

### Evolution of the magnitude and specificity of CD8^+ ^anti-HIV T cell responses

**Figure 1:**
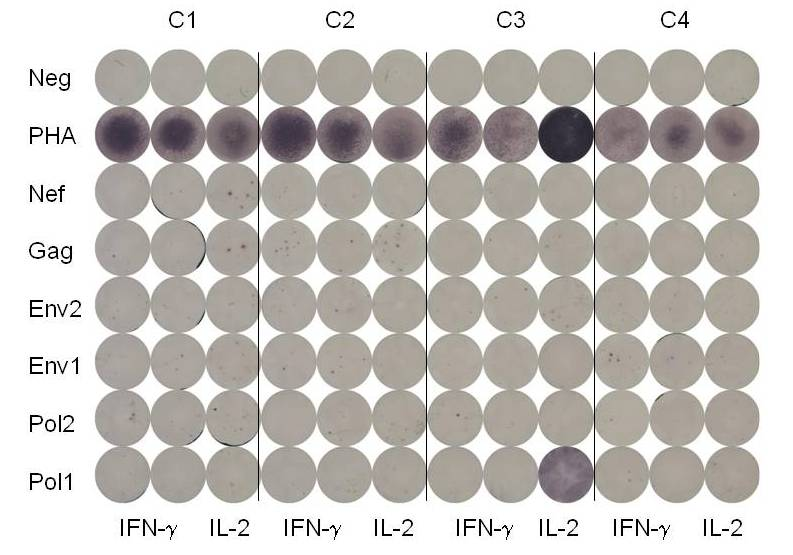
**Detection of HIV-specific IFN-γ and IL-2 spot forming cells (sfc) in uninfected controls**. Individual PBMC samples from four (C1, C2, C3 and C4) of six uninfected individuals tested by ELISPOT with medium alone (Neg), PHA and overlapping peptide pools from HIV Gag, Pol, Env and Nef for IFN-γ (duplicate) and IL-2 (single replicate) sfc are shown.

**Figure 2:**
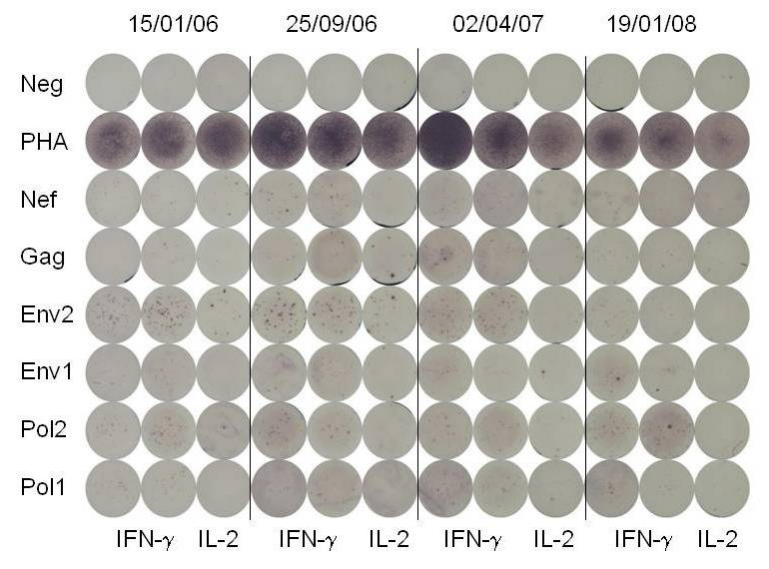
**Detection of HIV-specific IFN-γ and IL-2 spot forming cells in HIV-infected individuals with continuous suppression of viral replication**. Four PBMC samples taken on dates shown on top of the figure from a representative HIV-infected individual with continuous suppression of viral replication were tested as described in figure 1.

**Figure 3:**
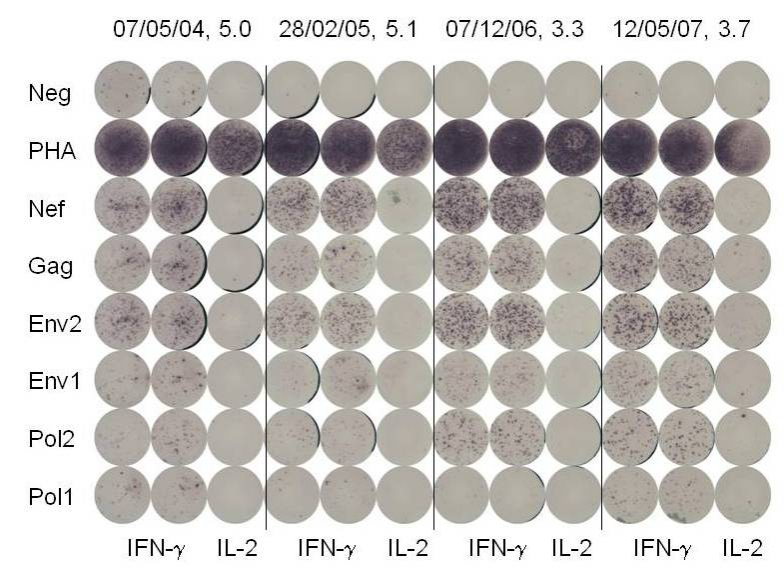
**Detection of HIV-specific IFN-γ and IL-2 spot forming cells in HIV-infected individuals with continuously detectable HIV replication**. Four PBMC samples taken from a representative HIV-infected individual with continuously detectable HIV replication on dates shown on top of the figure together with concurrent log_10 _copies HIV RNA/ml plasma were tested as described in figure 1.

**Figure 4:**
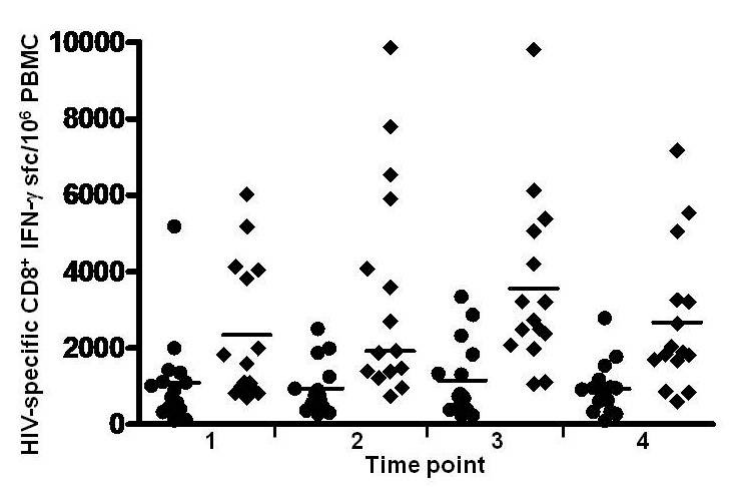
**Frequency of HIV-specific CD8^+ ^T cells producing IFN-γ in chronically infected individuals with ongoing HIV replication either detectable (◆) or suppressed below detectable levels (●)**. The total number of HIV-specific CD8^+ ^T cells producing IFN-γ/10^6 ^PBMC in response to Gag, Pol, Env and Nef peptide pools is shown for each individual at 4 serial time points with horizontal line through the group representing the median frequency.

**Figure 5:**
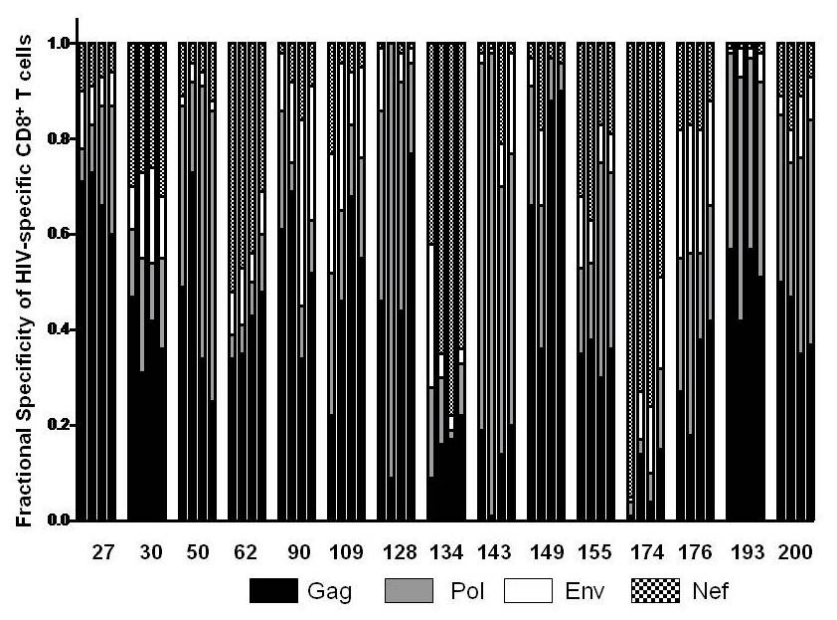
**Fractional specificity of the overall HIV-specific CD8^+ ^T cell response**. The fraction of the overall HIV specific CD8^+ ^T cell response attributed to each of the 4 major HIV proteins is shown by differential bar graph shading for individuals in the undetectable viral replication groups at each of 4 sequential time points tested.

ELISPOT testing of control PBMC (Figure 1) and 4 sequential PBMC samples from 15 individuals with persistently undetectable virus load (Figure 2) and 15 individuals with persistently detectable virus load (Figure 3) was carried out as shown. The cumulative number of INF-γ sfc/10^6 ^PBMC stimulated by the peptide pools representing HIV Gag, Pol, Env and Nef was taken as overall magnitude of the CD8^+ ^anti-HIV T cell response. Cells producing IFN-γ in response to the HIV peptides were almost exclusively CD8^+ ^as no increase over control values in the percentage of non-CD8^+ ^T cells producing IFN-γ was detectable by flow cytometry in any of the samples tested (data not shown). The fractional contribution of each individual HIV protein to overall magnitude indicated general specificity. The six uninfected controls had cumulative responses from 0-61 (median = 23) IFN-γ and 0-38 (median = 4) IL-2 sfc/10^6 ^PBMC in response to the HIV peptide pools, demonstrating the specificity and low background of the peptide ELISPOT assay. In the undetectable virus load group, overall magnitude of the CD8^+ ^anti-HIV T cell response ranged from 67 sfc/10^6 ^PBMC to 5166 sfc/10^6 ^PBMC (Figure 4). Median overall CD8^+ ^anti-HIV T cell response magnitude in this group remained consistent over the course of study, beginning at 874 and finishing at 895 sfc/10^6 ^PBMC, indicating no long-term decay in the magnitude of the CD8^+ ^anti-HIV T cell response, despite apparently effective antiretroviral therapy. Mean CD4^+ ^T cell count rose from 511 to 729/μl peripheral blood over the same time period (P = .002). If > 50% of the total INF-γ sfc were specific for 1 of the 4 major HIV proteins, we considered the response against this protein immunodominant. At the first study point Gag was the dominant CD8^+ ^T cell antigen for 6, Pol for 1, Nef for 2 and Env for 0 individuals in the undetectable virus group. This pattern persisted through the fourth study point with Gag immunodominant for 6, Pol for 2, Nef for 2 and Env for 0 individuals (Figure 5). In the absence of detectable HIV replication, several dramatic shifts in general antigenic dominance still occurred, from Gag to Pol (subject 50) and Pol to Gag (subject 128) (Figure 5). These cases of shifting immunodominance or epitope spreading together with persistently high HIV-specific CD8^+ ^T cell responses suggest immune stimulation by ongoing viral replication at levels undetectable by clinical virus load testing.

**Figure 6:**
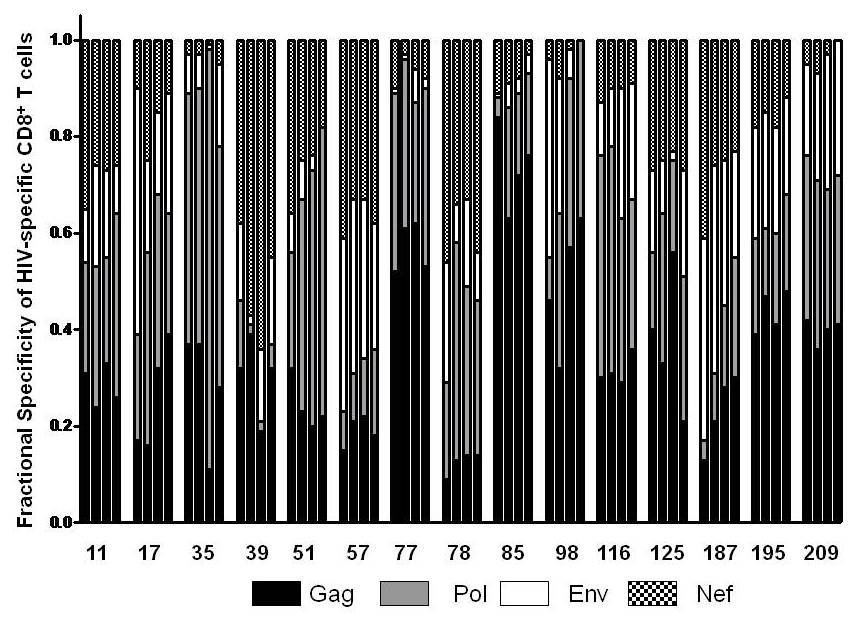
**Fractional specificity of the overall HIV-specific CD8^+ ^T cell response**. The fraction of the overall HIV specific CD8^+ ^T cell response attributed to each of the 4 major HIV proteins is shown by differential bar graph shading for individuals in the detectable viral replication groups at each of 4 sequential time points tested.

In the detectable virus load group, overall CD8^+ ^anti-HIV T cell response magnitude ranged from 608 to 9856 sfc/10^6 ^PBMC and increased (non-significant) from a median of 1587 to a median of 1889 sfc/10^6 ^PBMC over the course of the study (Figure 4). Median CD8^+ ^anti-HIV T cell response magnitude was significantly higher in the detectable virus load group than in controlled virus replication group (P < .0001). The mean CD4^+ ^T cell count was unchanged at 553 versus 524/μl peripheral blood at initial and final time points. Although these subjects had detectable virus replication throughout the study, all but three (187, 195 and 209) received partially suppressive antiretroviral therapy, either persistently or intermittently. At the first study point, Gag was the dominant CD8^+ ^T cell antigen for 2, Pol for 1, Nef for 1 and Env for 1 individual in the detectable virus load group. By the fourth study point, Gag was immunodominant for 3, Pol for 3, Nef for 0 and Env for 0 individuals (Figure 6).

### Evolution of memory status of CD8^+ ^anti-HIV T cell responses

**Figure 7:**
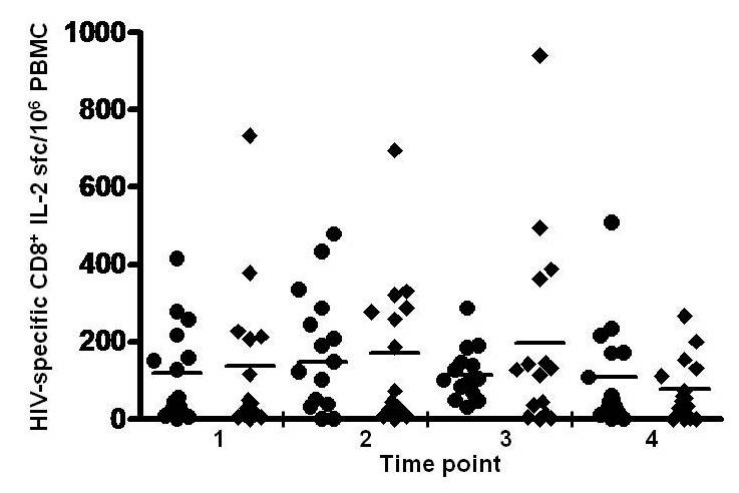
**Frequency of HIV-specific CD8^+ ^T cells producing IL-2 in chronically infected individuals with ongoing HIV replication either detectable (◆) or suppressed below detectable levels (●)**. The total number of HIV-specific CD8^+ ^T cells producing IL-2/10^6 ^PBMC in response to Gag, Pol, Env and Nef peptide pools is shown for each individual at 4 serial time points with horizontal line through the group representing the median frequency.

Central memory (Tcm) HIV-specific CD8^+ ^T cells are rare in progressive HIV infection and difficult to accurately enumerate by flow cytometry [10]. Therefore, we measured IL-2 producing cells by ELISPOT to estimate HIV-specific CD8^+ ^Tcm numbers (Figures 1, 2, 3). As expected, these were much less frequent than IFN-γ producing HIV-specific CD8^+ ^T cells. Although at lower absolute levels, response distribution across the 4 HIV antigens tested and overall expansion/contraction of the HIV-specific CD8^+ ^T cell IL-2 response generally mirrored that of the HIV-specific CD8^+ ^T cell IFN-γ response. For 12 individuals with the most prominent IL-2 sfc responses, individual peptides stimulating IL-2 were identified using peptide matrices from the appropriate peptide set. Optimal 9mers previously identified within the 15mers http://www.hiv.lanl.gov/content/immunology/ctl_search were then shown to stimulate IL-2 production, confirming that CD8^+ ^class I-restricted T cells were responsible for the IL-2 production. In the undetectable virus load group, the magnitude of the CD8^+ ^anti-HIV T cell IL-2 response ranged from 0 to 508 sfc/10^6 ^PBMC (Figure 7). Over the course of the study, the median overall CD8^+ ^anti-HIV T cell IL-2 response magnitude remained similar at 56 and 50 sfc/10^6 ^PBMC at time points 1 and 4 respectively. In the detectable virus load group, the magnitude of the CD8^+ ^anti-HIV T cell IL-2 response ranged from 0 to 940 sfc/10^6 ^PBMC and remained stable at a median of 43 and 55 sfc/10^6 ^PBMC at time points 1 and 4 respectively (Figure 7). There was no significant difference in the magnitude of CD8^+ ^HIV-specific IL-2 sfc/10^6 ^PBMC between groups and no significant changes in the magnitude of the response in either group over the course of the study. Viral replication reduced the fraction of HIV-specific CD8^+ ^Tcm with no impact on absolute number. Long term suppression of HIV replication had little impact on either the fraction or absolute number of CD8^+ ^HIV-specific IL-2 sfc.

**Figure 8:**
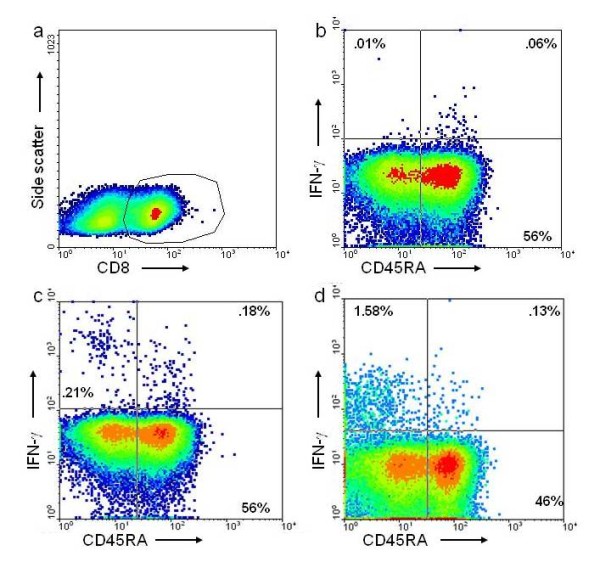
**CD45RA expression on CD8^+ ^HIV-specific T cells**. After overnight stimulation with HIV peptide pools, PBMC were stained with fluorescent antibodies against CD8, CD45RA and IFN-γ. (a) Lymphoid CD8^+ ^cells were gated for analysis of IFN-γ production and CD45RA expression. (b) Overnight incubation with medium alone. (c, d) Two representative samples are shown (50 and 116) with high and low fractions respectively, of CD45RA^+ ^HIV Pol-specific CD8^+ ^T cells producing IFN-γ following stimulation.

Since the contribution of CD45RA^+ ^naïve T cells to in vitro HIV-specific T cell responses is minimal, terminally differentiated HIV-specific CD8^+ ^effector cells can be distinguished by co-expression of intracellular IFN-γ and surface CD45RA following stimulation with HIV peptides. When cells were available and ELISPOT results indicated enough IFN-γ producing cells for phenotypic analysis of HIV-specific CD8^+ ^T cells by flow cytometry, we estimated the fraction of Ttd through CD45RA expression on HIV-specific CD8^+ ^T cells producing IFN-γ (Figure 8). In the undetectable virus load group, the CD45RA^+ ^percentage of the CD8^+ ^anti-HIV T cell response ranged from 13% to 84% (Table 1), but did not increase from a mean of 32% over the course of the study. In the detectable virus load group, the CD45RA^+ ^percentage of the CD8^+ ^anti-HIV T cell response ranged from 6% to 37% and fell from a mean of 23% to 17% over the course of the study. This decrease was not statistically significant. We observed a generally low percentage of CD8^+ ^anti-HIV T cells expressing CD45RA^+ ^throughout both groups of subjects, corroborating previous reports on impaired terminal differentiation of HIV-specific CD8^+ ^T cells [9,14].

## Discussion and Conclusions

The unusually low fractions of HIV-specific CD8^+ ^Tdt and Tcm detected in progressive infection suggest impaired differentiation may be a key factor in HIV-specific CD8^+ ^T cell dysfunction and progression to acquired immune deficiency syndrome (AIDS) [9,10,14]. Since CD8^+ ^T cells themselves are rarely HIV-infected, their impairment relates indirectly to HIV replication. Thus, whether HAART normalizes HIV-specific CD8^+ ^T cell differentiation in chronic infection remains unknown. In this study, we used overlapping peptides encompassing all of HIV Gag, Pol, Env and Nef to broadly examine HIV-specific CD8^+ ^T cell responses and address the impact successful antiretroviral suppression of HIV replication had on these responses. We compared evolution of the magnitude, specificity and character of HIV-specific CD8^+ ^T cell responses in two groups of individuals, both chronically infected with HIV, but distinguished by continuously detectable versus undetectable HIV replication over study periods from 2-9 years.

We found that the HIV-specific CD8^+^CD45RA^+ ^population, which includes Tdt, were relatively scarce throughout the detectable virus replication group and most of the undetectable virus replication group. While such paucity was previously described in studies focused on individual epitopes, the use of genome wide peptide pools herein clarifies its broader relevance to the HIV-specific CD8^+ ^T cell response [9,14]. The percentage of CD45RA^+ ^effectors, together with the absolute number of HIV-specific CD8^+ ^T cells remained relatively constant in both groups, consistent with establishment of stable ratios under chronic conditions of either antiretroviral suppression or HIV replication. While the overall similar character of HIV-specific CD8^+ ^T cell responses in both groups also supports the notion of intransigence to antiretroviral therapy, we did not compare the same individuals before and after introduction of successful antiretroviral therapy. Therefore, improvements in the character of HIV-specific CD8^+ ^T cell responses occurring early after onset of antiretroviral therapy would not have been detected in this study.

Although these responses would likely not be detectable by flow cytometry with individual peptides, we detected CD8^+ ^HIV-specific IL-2 responses in nearly half of the chronically infected individuals we tested by ELISPOT with peptide sets covering the entire HIV genome. The absolute and relative frequencies of HIV-specific CD8^+ ^T cells producing IL-2 were, nonetheless, very low in both groups, confirming that few, or in some cases, none of the HIV-specific CD8^+^CD45RA^- ^T cells were Tcm. The low numbers of HIV-specific IL-2 sfc indicate that only a very small fraction of the HIV-specific CD8^+^CD45RA^- ^IFN-γ producing T cells are Tcm. Therefore, prolonged or repeated expansion of HIV-specific effector cells skews the HIV-specific CD8^+ ^T cell population towards a predominant effector memory (Tem) phenotype and this skewing is not reversed by long-term suppression of HIV replication.

Identifying HIV-specific CD8^+ ^Tcm other than by expression of surface markers such as CCR7 could conceivably underestimate Tcm numbers, but proliferative function as indicated by IL-2 production may be a more meaningful discriminator of HIV-specific CD8^+ ^Tcm. The lower fraction of HIV-specific CD8^+ ^Tcm thus identified in the group with detectable HIV replication actually reflected higher absolute Tem numbers in this group, not fewer Tcm. Absolute Tcm numbers changed little over time in either group, implying a rough equilibrium of HIV-specific T cell subset distribution under relatively stable conditions of antiretroviral suppression or HIV replication. Thus, it appears that in stable settings of either viral replication or antiretroviral suppression, individuals reach a steady-state HIV-specific CD8^+ ^T cell response, analogous and related to virus load set-point [22]. This equilibrium would shift with acute expansion of Tem upon de novo viral replication or contraction of Tem with introduction of effective HAART. In our study, where initial samples were drawn at least 3 months after establishment of viral suppression or viral replication, the absolute magnitude, general specificity and subset distribution of the CD8^+ ^anti-HIV T cell response remained remarkably constant. This suggests an enormous reserve of CD8^+ ^HIV-specific effector cells, although previous or partially effective treatment probably also played a role in the durability of responses in our study group. In progressive infection, HIV-specific CD8^+ ^T cell activation, subset distribution and elimination maintains a set point that slowly decays towards true numerical deficits only in advanced infection. Set point maintenance in treated non-progressing subjects with undetectable viral loads indicates occult viral replication sufficient for continuous recruitment of relatively short-lived CD8^+ ^HIV-specific Tem or some residual conditioning of the immune system that perpetuates aberrant differentiation and subset distribution of HIV-specific CD8^+ ^T cells. Such low level replication, as was recently confirmed in both elite controllers and treated individuals with clinically undetectable virus loads, may be an important component of immune deficits that persist in chronic, treated HIV infection [19,23].

In humans, there are clear associations between initial suppression of viral replication and emergence of HIV-specific CD8^+ ^T cells as well as between advanced disease progression and decline of HIV-specific CD8^+ ^T cells [24-26]. In macaques, depletion of CD8^+ ^T cells in macaque models of HIV infection directly demonstrated the role that CD8^+ ^T cells play in lowering virus load in acute infection and delaying disease progression in chronic infection [27]. However, the coexistence of strong anti-HIV CD8^+ ^T cell responses with substantial levels of HIV replication that we and others have observed raises questions as to the long term contribution of these natural responses [28]. Some data indicate that clonal restriction of the HIV-specific CD8^+ ^T cell response is the earliest predictor of rapid disease progression, suggesting that the initial dynamics of CD8^+ ^T cell-mediated suppression of viral replication versus viral dissemination, diversification and immune destruction play a major role in determining the steady-state parameters of chronic infection [17,29]. Our data indicate that antiretroviral treatment in chronic HIV infection shifts the steady state towards slower decay and lower HIV-specific CD8^+ ^Tem frequencies without affecting the overall character of the HIV-specific CD8^+ ^T cell response. Without supplementary immunological conditioning to enhance the efficacy of HIV-specific immunity, the system will return to its original trajectory upon treatment failure or withdrawal.

In summary, we found no evidence that effective antiretroviral therapy influences any feature other than magnitude of HIV-specific CD8^+ ^T cell responses. Low frequencies of CD8^+ ^HIV-specific Tcm and low fractions of CD45RA^+ ^Tdt persisted over prolonged periods of effective HAART, suggesting that abnormal characteristics of the HIV-specific CD8^+ ^T cell response imprinted following acute infection endure immune restoration associated with HAART. However, none of the subjects in our study received antiretroviral therapy during acute infection, which may be more likely to influence HIV-specific CD8^+ ^T cell differentiation and subset distribution than treatment during chronic infection. Novel strategies addressing HIV-specific CD8^+ ^T cell response character will be required to achieve true immune restoration in chronic HIV infection.

## Competing interests

The authors declare that they have no competing interests.

## Authors' contributions

JP performed most of the ELISPOT and flow cytometry assays, coordinated sample selection and collated data. KZ and NAH performed additional ELISPOT assays. MEG processed and archived blood samples and collated clinical laboratory data. MDG designed the study, directed its conduct, interpreted data and prepared the manuscript. All authors read and approved the final manuscript.

## Pre-publication history

The pre-publication history for this paper can be accessed here:

http://www.biomedcentral.com/1471-2334/10/129/prepub
